# Phenotypic characterization of Landim and Pafúri goat populations in Southern Mozambique based on morpho-structural traits and zoometric indices

**DOI:** 10.1371/journal.pone.0331271

**Published:** 2026-06-17

**Authors:** Deiby T. Culhe, Matilde F. Matola, Elio Muatareque, Milton P. Morrombe, Matilde A. Manhique, Ramos J. Tseu, Abílio P. Changule, Maria da G. Taela, Custódio G. Bila, Manuel Garcia-Herreros

**Affiliations:** 1 Department of Animal Production and Food Technology, Faculty of Veterinary Medicine, Eduardo Mondlane University (UEM), Maputo, Mozambique; 2 Center for Genetic Resources and Animal Assisted Techniques (CRGTRA), Directorate of Animal Science (DCA), Agricultural Research Institute of Mozambique (IIAM), Matola, Mozambique; 3 Chobela Research Station, Centro Zonal Sul, Agricultural Research Institute of Mozambique (IIAM), Magude, Mozambique; 4 Department of Animal and Public Health, Faculty of Veterinary Medicine, Eduardo Mondlane University (UEM), Maputo, Mozambique; 5 Department of Research and Development, Intermed Mozambique Lda, Maputo, Mozambique; 6 Center of Excelence in Agri-Food Systems and Nutrition (CEAFSN) - Eduardo Mondlane University (UEM), Maputo, Mozambique; 7 Faculty of Veterinary Medicine and Animal Science, Save University (UniSave), Gaza Delegation, Chongoene, Mozambique; 8 National Institute for Agricultural and Veterinary Research (INIAV), Santarém, Portugal; 9 CIISA-AL4AnimalS, Faculty of Veterinary Medicine, University of Lisbon, Lisbon, Portugal; Ain Shams University Faculty of Agriculture, EGYPT

## Abstract

This study aimed to characterize the morpho-structural traits of indigenous goats reared at the Chobela Research Station located in Chobela neighbourhood, Magude District, southern Mozambique. A total of 137 goats were selected, comprising 77 Landim and 60 Pafúri animals. Qualitative (morphological) traits were assessed through visual inspection and summarized using frequencies and percentages, while quantitative (morphometric) traits were measured using a zoometric tape and analyzed using analysis of variance (ANOVA) at a 5% significance level. Due to low expected frequencies in some qualitative trait categories, inferential analysis (Chi-square test) was not performed, and these results are presented descriptively. In terms of morphological traits, all Pafúri goats exhibited a convex head profile, whereas Landim goats showed both convex (57.9%) and concave (42.1%) profiles. Approximately 75% of the goats presented a uniform coat colour pattern. Morphometric comparisons based on inferential analysis of female goats (n = 131) due to the limited number of males (n = 6), showed that Landim goats tended to have higher mean values for horn length (19.21 ± 0.83 cm), withers height (58.67 ± 1.20 cm), and body length (67.31 ± 1.73 cm), whereas Pafúri goats exhibited slightly larger head dimensions and thoracic perimeter. However, none of these differences were statistically significant (p > 0.05). Regarding zoometric indices, Landim goats tended to exhibit higher cephalic and thoracic indices, whereas Pafúri goats showed similar or slightly higher body, particularly among females. In contrast, Pafúri goats exhibited marginally higher proportionality indices, reflecting more elongated body proportions. Similarly, the small variations observed in zoometric indices did not reach statistical significance (p > 0.05). We hypothesized that, despite being reared under similar environmental conditions, Landim and Pafúri goat ecotypes would exhibit distinct morpho-structural characteristics due to differences in genetic background and adaptive history. These findings indicate that the morpho-structural differences between the two breeds are mainly descriptive rather than statistically supported. These results provide site-specific and preliminary baseline information for future genetic and phenotypic studies, thus may support conservation and sustainable utilization strategies for indigenous goat populations, particularly those reared under similar management and environmental conditions in southern Mozambique.

## Introduction

Goats (*Capra hircus*) are among the earliest domesticated livestock species and have supported human livelihoods for over 10,000 years [1]. Their remarkable adaptability enables them to thrive across a wide range of agroecological zones, from arid deserts to tropical forests and highlands [2]. Globally, more than 1,000 recognized breeds contribute to diverse production systems, offering meat, milk, fiber, skins, and fulfilling socio-cultural roles [3,4].

In many developing countries, particularly in sub-Saharan Africa, goats are a cornerstone of smallholder farming systems. They enhance food security, support income diversification, and serve critical functions in traditional ceremonies, dowries, and as a form of insurance against crop failure [5]. In Mozambique, the national goat population is estimated at approximately four million head, and has grown steadily over the past decade [6]. This increase is largely attributed to the species’ resilience to harsh climates, minimal input requirements, and their multiple functions in rural livelihoods [7,8]. Most goats are raised under extensive or semi-extensive systems characterized by low external inputs, high mobility, and limited selective breeding [9]. Despite their socio-economic and cultural importance, many of Mozambique’s indigenous goats remain poorly characterized and lack targeted conservation programs. Landim and Pafúri populations are classified as ecotypes, as they lack clearly defined breeding structures and uniform selection criteria. Both ecotypes are traditionally raised in the southern provinces and are valued for their adaptability and meat quality. However, unregulated crossbreeding and the absence of structured breeding schemes have raised concerns about potential genetic erosion and loss of local adaptation [10,11]. At present, the conservation status of these ecotypes is considered data-deficient, as no formal *in situ* or *ex situ* conservation initiatives are currently in place [12].

Earlier studies have provided preliminary insights into the genetic and morphological diversity of Mozambican goats. Garrine et al. [10] demonstrated genetic differentiation between the Landim and Pafúri populations using microsatellite markers, confirming distinct genetic signatures between ecotypes. Magaço and Felimone [13] applied principal component analysis (PCA) to body measurements of Mozambican local goats and identified key conformation axes that explained most of the observed phenotypic variability. Together, these studies indicate considerable within-population diversity and highlight the need for broader, integrated phenotypic and genomic assessments across regions [14,15].

In this context, morpho-structural traits refer to the qualitative physical features that define the animal’s overall body conformation, such as head profile, horn orientation, ear type, and coat pattern [16,17]. Morphometric traits, on the other hand, are the quantitative linear measurements of the body such as head length, body length, thoracic perimeter, and withers height that provide numerical indicators of size and proportions [8,18]. Meanwhile, breed-specific traits encompass distinctive morphological or genetic characteristics unique to each breed, which allow for breed identification and differentiation [18,19]. Together, these parameters provide complementary information for a robust phenotypic and functional characterization of indigenous goat breeds. Such efforts are fundamental to the development of sustainable breeding programs and the prevention of genetic erosion.

Landim goats have short-eared heads that are concave in females and slightly convex in males; horns curve backward in both sexes but are heavier in males [8,10]. All males and 12% of females carry beards [10,13]. Coats vary in colour (black, white, brown, or spotted), ears are erect, and the hair is short and fine [10,20]. Bucks sport a short, dense mane along the backline and reach about 45 kg at two years, while does average 35 kg [8,10,13,20]. Pafúri goats display a convex head profile with divergent, well-developed horns in males and smaller, scimitar-shaped horns in females [20]. Ears are medium-length, sometimes trimmed, with rounded tips. Both sexes have beards, a strong, well-set neck, straight back, and well-muscled limbs [13]. Adult males weigh roughly 60 kg and females about 43 kg [6,13,16,17]. This study aimed to characterize the morpho-structural traits of two indigenous goat ecotypes Landim and Pafúri reared at the Chobela Research Station in Magude District, southern Mozambique. We hypothesized that, despite sharing the same environment, Landim and Pafúri goats would display distinct morpho-structural profiles resulting from their different genetic backgrounds and adaptive historiesdes.

### Materials and methods

**Fig 1:**
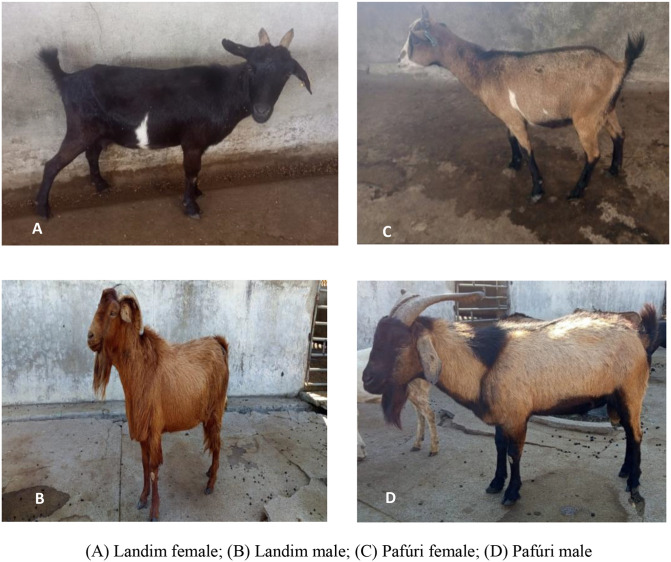
Photographs of the two indigenous goat ecotypes included in the study. (A) Landim female; (B) Landim male; (C) Pafúri female; (D) Pafúri male.

### Ethical statement

The study was conducted in accordance with the national and institutional guidelines for the care and use of animals for scientific purposes, and followed the ethical principles outlined in the Code of Ethics for Animal Experiments and the ARRIVE guidelines (https://arriveguidelines.org). The research protocol was reviewed and approved by the Bioethics Committee for the Use of Experimental Animals at Eduardo Mondlane University, Mozambique (Approval Code: CBUAE-112-UEM-MZ, approval date: 20 March 2023).

Field site access and animal handling were authorized by the Directorate of Animal Science of the Agricultural Research Institute of Mozambique (IIAM), which manages the Chobela Research Station where the study was conducted. The work took place entirely within institutional research premises, and therefore, no additional government or private permits were required.

### Study location and animal management

This study was conducted at the Chobela Research Station (CZC), located in Chobela neighborhood, Magude District, southern Mozambique. The district lies between latitudes 24°59′20″ S and longitudes 32°45′10″ E, and is bordered by the districts of Chókwè and Bilene to the north, Moamba to the south, Manhiça to the east, and the Republic of South Africa to the west [21]. The area supports both private and communal livestock systems, with the main species being cattle, goats, pigs, sheep, and poultry (primarily indigenous chickens). The climate is classified as dry subtropical (Köppen classification), with average annual temperatures ranging from 22 to 24°C and an average annual rainfall of approximately 630 mm. There are two distinct seasons: a hot and rainy season from October to March, which accounts for around 80% of the annual precipitation, and a cooler, dry season from April to September [21].

The goats were maintained under an extensive production system using natural pasture feeding. To minimize the effect of pregnancy on morphometric measurements, only non-pregnant adult does aged ≥ 2 years were included in the study to reduce the influence of growth-related variation on morphometric traits. However, precise chronological age data were not consistently available; therefore, age classes or age as a covariate could not be incorporated into the statistical analyses. The reproductive status of each female was assessed through visual inspection for abdominal enlargement, palpation, and verification of herd breeding records. Does with any indication of pregnancy were excluded. While very early pregnancies may not have been detected, such cases are expected to be rare and unlikely to influence the overall characterization. For the study, a total of 137 breeding goats were selected, comprising two indigenous ecotypes, Landim (n = 77) and (n = 60). The sampling followed a census approach, whereby all available adult animals meeting the inclusion criteria were evaluated, rather than random sampling. The photographs of the Landim and Pafúri are shown in Fig 1.

This study was conducted using the full populations of Landim and Pafúri goats maintained at the Chobela Research Station for more than 50 years. This station represents a nucleus herd for these two indigenous ecotypes. Thus, all available adult animals that met the inclusion criteria (n = 137; 131 females and 6 males) were evaluated. The sampling framework was therefore determined by the herd composition at the station rather than by proportional national herd size. Although the FAO [2,14] guidelines recommend multi-herd sampling and a minimum of 100 females and 50 males per breed population for phenotypic characterization, this study aimed to provide a controlled comparative assessment under uniform management and environmental conditions. Accordingly, results should be interpreted as a site-specific baseline for these ecotypes.

### Data collection

Data collection included the recording of morphological characteristics (qualitative) and linear/ morphometric body measurements (quantitative traits) of Landin and Pafúri goats according to FAO [14].

### Qualitative traits

Qualitative (morphological) traits were recorded through direct visual inspection according to guidelines for phenotypic characterization [14]. The traits assessed included head characteristics (head size and shape, and profile), beard presence or absence, horn characteristics (presence, size, orientation, and shape), ear characteristics (size, shape, and position), dorsal line presence, limb type (muscular, intermediate, or fine), coat characteristics (hair length and coat colour pattern), and overall body conformation classified as light, intermediate, or heavy.

### Quantitative traits

Quantitative traits (morphometric measurements) were obtained through a linear body measurement using a standard zoometric measuring tape. These measurements included head length, head width, ear length, horn length, withers height, thoracic perimeter, and body length, following proper physical restraint of each animal to ensure both data accuracy and animal and handler safety. Restraint was carried out gently, typically with the aid of halters or hand-held by trained personnel, to minimize movement and stress during measurement. This procedure aligns with the guidelines recommended for the phenotypic characterization of animal genetic resources following the FAO guidelines [2,14]. Specifically, head length was measured from the occipital crest to the tip of the muzzle; head width as the maximum distance between the zygomatic arches; ear length from the base to the tip of the pinna; horn length from the base at the skull to the tip along the outer curvature; withers height from the highest point of the withers to the ground; thoracic perimeter as the circumference of the chest just behind the forelegs; and body length from the scapulo-humeral joint (point of shoulder) to the ischial tuberosity (pin bone). Zoometric indices were calculated using standard formulas to evaluate somatic development and infer functional conformation (Table 1). The following indices were computed as described by FAO [11,22]– 24].

**Table 1:** summary of calculated zoometric indices using formula.

Index	Formula	Anatomical measures used
Body Index (BI)	(BL / TP) × 100	Body Length (BL), Thoracic Perimeter (TP)
Cephalic Index (CI)	(HW / HL) × 100	Head Width (HW), Head Length (HL)
Thoracic Index (TI)	(WH / TP) × 100	Withers Height (WH), Thoracic Perimeter (TP)
Proportionality Index (PI)	(WH / BL) × 100	Withers Height (WH), Body Length (BL)
Lateral Body Index (LBI)	BL / HW	Body Length (BL), Head Width (HW)
Anamorphosis Index (AI)	TP² / WH	Thoracic Perimeter (TP), Withers Height (WH)
Pelvic Index (PeI)	(HW / HL) × 100	Head Width (HW), Head Length (HL)
Dactyl-thoracic Index (DTI)	(HW / TP) × 100	Head Width (HW), Thoracic Perimeter (TP)
Dactyl-costal Index (DCI)	(HW / BL) × 100	Head Width (HW), Body Length (BL)
Transverse Pelvic Index (TPI)	(HW / WH) × 100	Head Width (HW), Withers Height (WH)
Longitudinal Pelvic Index (LPI)	(HL / WH) × 100	Head Length (HL), Withers Height (WH)

These indices were used to classify the animals according to their body conformation brevilinear, mesolinear, or longilinear and to infer their potential for meat production, particularly regarding compactness, proportionality, and skeletal development.

### Statistical analysis

Morphological traits were summarized using frequencies and percentages and due to low expected frequencies in several categories, they were presented descriptively. The effects of ecotypes and sex, as well as their interaction, were initially assessed using a two-way analysis of variance ANOVA. The following model was applied:


$$\begin{array}{c} \text{y }=\;\mu \;+\text{ breed }+\text{ sex }+\text{ breed }\times \text{ sex }+\text{ e} \end{array}$$

where:

- y = observed trait
- μ = overall mean
- Breed = fixed effect of ecotype
- Sex = fixed effect of sex
- Breed × Sex = interaction effect
- e = residual error

The interaction effect of ecotypes and sex was not significant (p > 0.05) for any morphometric trait, thus the final analysis was restricted to the main effect of breed using data from females only, due to the small number of males in the dataset. Tukey’s post hoc test was used for mean comparisons. Assumptions of normality and homoscedasticity were verified using the Shapiro-Wilk and Levene’s tests, respectively. All statistics analysis were performed in RStudio version 2023.06.1 (R Foundation for Statistical Computing, Vienna, Austria) with functions including aov() (base), shapiro.test() (base), and levene Test() from the car package. Statistical significance was set at p ≤ 0.05.

## Results

### Qualitative characteristics

The distribution of coat colours variant and pattern among the two goat populations is summarized in Table 2. Three coat colour variants, black, brown (light to dark), and white, were observed in both Landim and Pafúri goat populations, with brown being the most frequent variant overall. The distribution of coat colour variants was similar between ecotypes.

**Table 2:** estimated frequencies and percentages of coat colour variants and coat colour pattern in Mozambican Indigenous Goat Breeds (Landim and Pafúri).

Coat color	Landim (n)	Landim (%)	Pafúri (n)	Pafúri (%)	Total (n)	Total (%)
**Type**						
Black	18	24.0	12	20.0	30	22.2
Brown (light/dark)	21	28.0	18	30.0	39	28.9
White	12	16.0	10	16.7	24	16.3
Total	77	100	60	100	137	100
**Pattern**						
Uniform (simple)	56	75.0	45	75.0	101	75.0
Spotted / Mixed	19	25.0	15	25.0	36	25.0
Total	77	100	60	100	137	100

The qualitative morphological characteristics of the two breeds are presented in Table 3. Landim goats exhibited two distinct head profiles convex (57.9%) and concave (42.1%) whereas all Pafúri goats displayed a convex profile. Ear orientation differed completely between breeds: Landim goats had erect ears, while Pafúri goats had pendulous ears. All goats were horned, but horn shape and orientation varied, where 36.8% of Landim goats had horns curved backward, compared to 82.4% of Pafúri goats with laterally oriented, spiral-shaped horns. Beard presence was more frequent in Pafúri (36.7%) than in Landim goats (24%). Coat type was predominantly short in both breeds, 70% in Landim, and 60% in Pafúri.

**Table 3:** morphological characteristics observed in the Landim and Pafúri goat ecotypes.

Category	Variable	Modality	Landim (%)	Pafúri (%)
**Head**	Size	Small	28.0	15.0
		Medium	42.7	55.0
		Large	29.3	30.0
	Shape	Short	34.7	20.0
		Normal	41.3	45.0
		Long	24.0	35.0
	Profile	Concave	42.1	0.0
		Straight	0.0	0.0
		Convex	57.9	100.0
**Beard**	Presence	Present	24.0	36.7
		Absent	76.0	63.3
**Horns**	Presence of horns	Present	100.0	100.0
	Horn size	Small	27.0	15.0
		Medium	49.0	52.0
		Large	24.0	33.0
	Horn orientation	Curved backward	36.8	17.6
		Curved upward	36.8	0.0
		Lateral	26.3	82.4
	Horn shape	Straight	36.8	0.0
		Spiral	31.6	82.4
		Curved	31.6	17.6
**Ears**	Ear size	Small	20.0	10.0
		Medium	47.0	58.0
		Large	33.0	32.0
	Shape	Erect	100.0	0.0
		Pendulous	0.0	100.0
	Position	Vertical	92.0	40.0
		Horizontal	8.0	60.0
**Dorsal Line**	Presence	Present	71.0	85.0
		Absent	29.0	15.0
**Extremities**	Type of limbs	Muscular	43.0	47.0
		Intermediate	35.0	30.0
		Fine	22.0	23.0
**Coat**	Hair type	Short	70.0	60.0
		Medium	25.0	30.0
		Long	5.0	10.0
	Pattern	Simple (uniform)	75.0	75.0
		Irregular (spotted)	25.0	25.0
**Apparent Weight**	Body conformation	Heavy	22.0	38.0
		Intermediate	50.0	47.0
		Light	28.0	15.0

### Quantitative characteristics

The mean values for linear body measurements are shown in Table 4. No statistically significant differences were detected between breeds (p > 0.05). However, Landim females tended to have slightly higher mean values for horn length (19.21 ± 0.83 cm), withers height (58.67 ± 1.20 cm), and body length (67.31 ± 1.73 cm), whereas Pafúri females exhibited marginally larger head dimensions (head length 17.43 ± 0.75 cm; head width 11.17 ± 0.59 cm) and thoracic perimeter (67.20 ± 2.27 cm).

**Table 4:** Morphometric characteristics observed in Landim and Pafúri goat.

Trait	Landim (cm)	Pafúri (cm)	p-value
	Female	Female	
Head length (HL)	17.3 ± 0.65	17.4 ± 0.75	0.856
Head width (HW)	11.1 ± 0.57	11.2 ± 0.59	0.894
Ear length (EL)	13.1 ± 0.52	13.2 ± 0.49	0.900
Horn length (HL)	19.2 ± 0.83	19.0 ± 0.80	0.856
Withers height (WH)	58.7 ± 1.20	58.5 ± 1.66	0.946
Thoracic perimeter (TP)	66.9 ± 1.25	67.2 ± 2.27	0.924
Body length (BL)	67.3 ± 1.73	67.1 ± 6.11	0.972

HL = Head length; HW = Head width; EL = Ear length; HoL = Horn length; WH = Withers height; TP = Thoracic perimeter; BL = Body length. Values are presented as mean ± standard deviation (SD). No significant differences (p > 0.05).

The calculated zoometric indices are presented in Table 5. None of the indices differed significantly between breeds (p > 0.05). Landim goats showed slightly higher mean values for body index (99.45 ± 3.16), cephalic index (64.10 ± 4.09), and thoracic index (114.10 ± 4.72). Conversely, Pafúri goats exhibited marginally higher proportionality and anamorphosis indices (87.22 ± 8.33 and 78.28 ± 5.56, respectively). Based on standard classification criteria, Landim goats were characterized as mesolinear, while Pafúri goats were brevilinear in conformation.

**Table 5:** Zoometric index traits in Landim and Pafúri goats.

Zoometric Index	Landim		Pafúri		p-value
	Male	Female	Male	Female	
Body Index (BI)	99.9 ± 2.28	99.5 ± 3.16	99.8 ± 9.80	99.7 ± 9.73	0.829
**Cephalic Index (CI)**	64.6 ± 3.59	64.1 ± 4.09	63.9 ± 5.28	64.1 ± 4.37	1.000
**Thoracic Index (TI)**	114.1 ± 5.62	114.1 ± 4.72	115.0 ± 5.29	114.8 ± 5.39	0.532
**Proportionality Index (PI)**	89.7 ± 1.86	87.7 ± 2.86	88.2 ± 8.34	87.2 ± 8.33	0.691
**Lateral Body Index (LBI)**	6.1 ± 0.45	6.1 ± 0.37	6.1 ± 0.41	6.0 ± 0.34	0.174
**Anamorphosis Index (AI)**	77.4 ± 3.88	76.4 ± 4.28	78.5 ± 5.60	78.3 ± 5.56	0.108
**Pelvic Index (PI)**	63.1 ± 4.21	64.1 ± 3.89	64.1 ± 4.20	64.1 ± 4.18	0.948
**Dactyl-thoracic Index (DTI)**	17.5 ± 1.17	16.5 ± 1.11	16.5 ± 1.12	16.4 ± 1.09	0.606
**Dactyl-costal Index (DCI)**	16.5 ± 1.20	16.4 ± 1.10	16.3 ± 1.14	16.2 ± 1.04	0.392
**Transverse Pelvic Index (TPI)**	19.0 ± 1.42	18.9 ± 1.39	19.0 ± 1.54	18.9 ± 1.34	0.778
**Longitudinal Pelvic Index (LPI)**	30.4 ± 2.30	29.4 ± 2.02	29.8 ± 2.18	29.7 ± 2.08	0.396

Values are presented as mean ± standard deviation. TP: Thoracic perimeter; BL: Body length; WH: Withers height; HL: Head length; HW: Head width. BI: Body Index; CI: Cephalic Index; TI: Thoracic Index; PI: Proportionality Index; LBI: Lateral Body Index; AI: Anamorphosis Index; PeI: Pelvic Index; DtI: Dactyl-thoracic Index; DcI: Dactyl-costal Index; TPI: Transverse Pelvic Index; LPI: Longitudinal Pelvic Index. No significant differences (p > 0.05).

## Discussion

This study was based on the assumption that, although the Landim and Pafúri goat breeds were raised under similar environmental and management conditions, they would show morpho-structural differences due to their distinct genetic backgrounds and adaptive histories [10]. However, the results revealed a high degree of similarity between the two ecotypes, particularly in morphometric traits, for which no statistically significant differences were observed. Overall, the morphometric and zoometric analyses revealed no statistically significant differences between Landim and Pafúri goats and should be interpreted as descriptive trends rather than evidence of phenotypic differentiation.

Despite this overall similarity in morphometric traits, certain qualitative morphological characteristics suggest some degree of differentiation between breeds. For example, all Pafúri goats displayed a convex head profile, whereas Landim goats showed greater qualitative variation in head profile, horn orientation and shape. Despite that these qualitative differences are subtle, they may reflect breed-specific phenotypic adaptive features shaped by management and crossbreeding histories [15,19].

The non-observed morphometric divergence between the Landim and Pafúri populations possibly indicates substantial phenotypic overlap. This may be linked to widespread uncontrolled mating and the lack of structured breeding programs. These findings emphasize the limitations of relying solely on phenotypic characterization to distinguish local breeds, especially in low-input systems with little reproductive control.

The phenotypic variation observed in Landim and Pafúri goats reflects the richness and complexity of indigenous goat populations in Mozambique. The wide range of horn orientations and diverse coat patterns among Landim goats indicates substantial intra-breed variability. The phenotypic variation observed in Landim and Pafúri goat ecotypes likely reflects a combination of historical and ongoing gene flow between populations, as well as shared ancestry prior to the establishment of the nucleus herds. These processes are consistent with production systems commonly found in sub-Saharan Africa, where communal grazing and limited control over mating facilitate continuous genetic exchange among populations. As a result, weak phenotypic differentiation and overlapping breed boundaries are frequently observed in indigenous goat populations under such management conditions [2,23]. Additionally, the long-term maintenance of genetically heterogeneous breeding stocks at the research station may have further contributed to the observed phenotypic overlap [26,27].

The uniform convex head profile and predominance of spiral-shaped horns in Pafúri goats are consistent with their lineage as crossbreeds between Boer bucks and indigenous does [20]. However, the limited phenotypic differentiation between the two breeds observed in this study may indicate ongoing gene flow and the absence of structured selection programs which is a concern also noted in phenotypic assessments of indigenous goats across Eastern and Southern Africa [15].

The lack of statistically significant differences in linear body measurements between the breeds suggests a high degree of morphological overlap. Even so, trends such as greater horn length and withers height in Landim goats, and broader heads and larger thoracic perimeters in Pafúri goats, may reflect adaptive responses to ecological niches and local selection pressures [3,25]. Morphological traits in goats are known to be influenced by environmental and climatic factors, which shape phenotypic variation through both natural and human-driven selection. Variation in body size and conformation, including withers height and thoracic development, has been associated with adaptation to different agroecological zones, where larger body dimensions may enhance mobility, foraging efficiency, and thermoregulation under specific environmental conditions. Similarly, structural conformation traits such as body depth, girth, and trunk dimensions have been shown to vary across locations and are considered indicators of environmental adaptation and production potential in indigenous goat populations [28]. Horn characteristics, including size and shape, may also reflect adaptive and functional roles related to thermoregulation, social behavior, and defense, which are influenced by ecological pressures and management systems. More broadly, phenotypic traits in goats are shaped by climate-driven selection processes, where environmental factors such as temperature, feed availability, and terrain contribute to observable differences in morphology across populations [29]. Therefore, although the differences observed in this study were not statistically significant, the directional trends in certain morphometric traits may still reflect underlying adaptive responses to local environmental conditions and historical selection pressures.

This morphometric stability in adult animals is expected, as body measurements tend to stabilize after maturity. It reflects the combined influence of genetic potential and environmental adaptation [10,22]. Nonetheless, differences in certain traits may hold adaptive or functional value within local production systems and should be considered in community-based selection programs [4,26].

Zoometric indices provide useful insights into animal conformation and production potential. In this study, Landim goats exhibited higher body, cephalic, and thoracic index values, while Pafúri goats showed slightly higher proportionality index values. These findings support the classification of Landim goats as mesolinear and Pafúri goats as brevilinear, corresponding to moderate and compact body frames, respectively, which are both suitable for meat production [24].

Although zoometric indices are valuable for comparative analysis, the high morphological similarity between the two breeds suggests that morphometric data alone are insufficient for reliable breed differentiation. Recent studies highlight the importance of integrating morphometric, genomic, and environmental data to achieve comprehensive characterization and sustainable management of indigenous livestock breeds [18].

Uncontrolled mating systems remain a major constraint for breed conservation in low-input livestock systems like in Chobela Research Station. In such contexts, the absence of mating control and structured breeding programs accelerates genetic erosion and threatens the long-term viability of local genetic resources [27]. These observations are consistent with our findings, where both ecotypes showed overlapping morphometric values despite minor qualitative differences.

### Limitations and future perspectives

This study provides baseline phenotypic information for Landim and Pafúri goats maintained at the Chobela Research Station; however, several limitations are acknowledged. The limited number of males restricted sex-based analyses, and the absence of precise age records precluded age-adjusted statistical modelling. Additionally, the relatively small sample size limited inferential analysis of qualitative traits. Future studies should incorporate larger and sex-balanced data, and detailed age recording, as well as employ genomic approaches to better elucidate the genetic basis of phenotypic variation and population structure in Landim and Pafúri goat populations.

## Conclusion

This study provides baseline phenotypic characterization of Landim and Pafúri goats maintained under uniform environmental and management conditions, although reproduction within the herd occurs under uncontrolled mating without structured breeding programs. The analysis revealed a high degree of phenotypic overlap between the two populations, with most morphometric and zoometric traits showing no statistically significant differences. Although minor descriptive variations were observed, these were not sufficient to support clear phenotypic differentiation between breeds. The findings highlight the influence of shared management, environmental conditions, and historical gene flow on shaping observable morphology and underscore the importance of cautious interpretation of phenotypic variation in the absence of strong statistical support.

## Supporting information

S1 File Dataset used for the phenotypic characterization of Landim and Pafúri goat populations in southern Mozambique. The file contains the raw data used for the analysis of qualitative morphological traits, morphometric measurements, and calculated zoometric indices of the goats included in the study.
(XLSX)
